# Response of Downy Oak *(Quercus pubescens* Willd.) to Climate Change: Transcriptome Assembly, Differential Gene Analysis and Targeted Metabolomics

**DOI:** 10.3390/plants9091149

**Published:** 2020-09-04

**Authors:** Jean-Philippe Mevy, Beatrice Loriod, Xi Liu, Erwan Corre, Magali Torres, Michael Büttner, Anne Haguenauer, Ilja Marco Reiter, Catherine Fernandez, Thierry Gauquelin

**Affiliations:** 1 CNRS, Aix-Marseille University, Avignon University, IRD, IMBE, 13331 Marseille, France; anne.haguenauer@criobe.pf (A.H.); catherine.fernandez@imbe.fr (C.F.); thierry.gauquelin@imbe.fr (T.G.); 2 TGML-TAGC—Inserm UMR1090 Aix-Marseille Université 163 avenue de Luminy, 13288 Marseille, France; beatrice.loriod@inserm.fr (B.L.); magali.torres@univ-amu.fr (M.T.); 3 CNRS, Sorbonne Université, FR2424, ABiMS platform, Station Biologique, 29680 Roscoff, France; xi.liu@sb-roscoff.fr (X.L.); erwan.corre@sb-roscoff.fr (E.C.); 4 Metabolomics Core Technology Platform Ruprecht-Karls-University Heidelberg Centre for Organismal Studies (COS) Im Neuenheimer Feld 360, 69120 Heidelberg, Germany; michael.buettner@cos.uni-heidelberg.de; 5 Research Federation ECCOREV FR3098, CNRS, 13545 Aix-en-Provence, France; ilja.reiter@web.de

**Keywords:** transcriptome analysis, metabolism, drought, *Quercus pubescens*, adaptation, RNASeq

## Abstract

Global change scenarios in the Mediterranean basin predict a precipitation reduction within the coming hundred years. Therefore, increased drought will affect forests both in terms of adaptive ecology and ecosystemic services. However, how vegetation might adapt to drought is poorly understood. In this report, four years of climate change was simulated by excluding 35% of precipitation above a downy oak forest. RNASeq data allowed us to assemble a genome-guided transcriptome. This led to the identification of differentially expressed features, which was supported by the characterization of target metabolites using a metabolomics approach. We provided 2.5 Tb of RNASeq data and the assembly of the first genome guided transcriptome of *Quercus pubescens*. Up to 5724 differentially expressed transcripts were obtained; 42 involved in plant response to drought. Transcript set enrichment analysis showed that drought induces an increase in oxidative pressure that is mitigated by the upregulation of ubiquitin-like protein protease, ferrochelatase, oxaloacetate decarboxylase and oxo-acid-lyase activities. Furthermore, the downregulation of auxin biosynthesis and transport, carbohydrate storage metabolism were observed as well as the concomitant accumulation of metabolites, such as oxalic acid, malate and isocitrate. Our data suggest that early metabolic changes in the resistance of *Q. pubescens* to drought involve a tricarboxylic acid (TCA) cycle shunt through the glyoxylate pathway, galactose metabolism by reducing carbohydrate storage and increased proteolytic activity.

## 1. Introduction

Global change refers to a wide range of global phenomena such as warming, increased sea levels and extreme weather events. This includes significant changes in either the mean state of the climate or in its variability, persisting for an extended period as defined by the United Nations Intergovernmental Panel on Climate Change [1]. Different biomes are impacted in different ways, but the consequences in terms of ecosystem functioning and ecosystem services are far from being well understood.

The Mediterranean basin stands at the crossroads of the influences of the arid climate of northern Africa and the temperate climate of central Europe, and this raises the question of the vulnerability of this region to climate change variables. The region accounts for about 25,000 autochthon plant species, among which more than half are endemic. The Mediterranean basin was thus recognized as one of the first 25 global biodiversity hotspots [2]. At the same time, annual rain fall is projected to decline by 20–30% by the end of this century [3,4] which confirms the urgency of setting up conservation strategies in relation to the vulnerability of these ecosystems to drought.

The limits of the distribution areas of the Mediterranean basin will be modified by climate change depending on the drought tolerance range of species. The most sensitive species are therefore those with specific tolerance thresholds for precipitation and other factors. However, little is known about how these species will cope with the predicted climate change.

The genus *Quercus* (Fagaceae, family) is one of the most important taxa of woody angiosperms in the northern hemisphere. In the Mediterranean basin, *Quercus pubescens,* or downy oak, is often found in coastal plains to about 1300 m altitude. It is a heliophilous and thermophilous species and is well adapted to moderate summer drought stress, although it avoids continental locations subjected to more frequent drought events.

Several studies have been carried out based on the tolerance of *Q. pubescens* to drought ranging from morphoanatomy to physiology. For instance, in drought-stressed individuals, a significant earlier senescence and increased amounts of soluble sugars in the leaves were observed [5]. This high sugar content is in accordance with the capacity of *Q. pubescens* to maintain much higher photosynthetic potential when compared to other species, such as *Quercus ilex, Pinus halepensis* and *Arbutus unedo* [6]. The capacity of *Q. pubescens* to control the antioxidant system response to drought was also demonstrated [7]. In general, the response of early plants to water deficit involves complex mechanisms related to the reduction in growth and stomatal conductance with the concomitant decrease in photosynthesis activity [8,9] in order to prevent excessive water loss and tissue oxidation. Subsequently, depending on the duration and intensity of water stress, specific metabolic processes are activated on the basis of transcription of several categories of genes. Overall, it is considered that the genes expressed in response to drought can be divided into two broad categories: those expressing stress proteins and those encoding regulatory ones [10]. Despite these advances, the genetic and metabolic basis of the adaptation of plants to water stress still remain unclear because of the complexity of the metabolic networks and the specificity of ecotypes.

The onset of NGS (next-generation sequencing) technologies as well as metabolomic tools offer new opportunities to explore the response of the whole genome to environmental constrains. The de novo trancriptome assembly of *Q. pubescens* plantlets cultivated in greenhouse conditions led to the identification of several genes associated with drought avoidance [11]. However, in controlled environments, plant growth often does not totally reflect the conditions experienced in their natural setting, especially with regards to the prevailing light conditions, biotic interactions and soil conditions. In addition, transcriptomic analyses should be associated with ecophysiological and metabolic data for a complete description of the response of plants to environmental factors. Indeed, few studies have attempted to analyze transcriptome from plants grown in situ and even fewer have underpinned these studies with thorough in situ physiological characterization [12]. To address these requirements, the concept of ecological genomics has been proposed as an integrative discipline aimed at understanding the genetic mechanisms in relation to the responses of organisms to the conditions of their natural environment [13]. Ecological genomics thus associate the study of ecophysiological components to those of the transcripts, proteins and metabolites as to understand gene function and coordination between cellular processes and biochemical pathways.

The aim of this in situ study was first to test the hypothesis of plant–climate coevolution through the transcription profile for drought response. Second, a multi-omic approach linking transcriptomics and metabolomics was carried out to bridge the gap between gene expression, metabolic and ecophysiological responses, and to provide new insights into plant water-stress-dependent metabolic networks. For these purposes, a robust transcriptome was assembled, improving also the scarcity of the genomic resource of *Q. pubescens* by generating a clean 50 Gb RNA-Seq dataset.

## 2. Materials and Methods

### 2.1. Climate Change Simulation in Oak Forest

**Figure 1:**
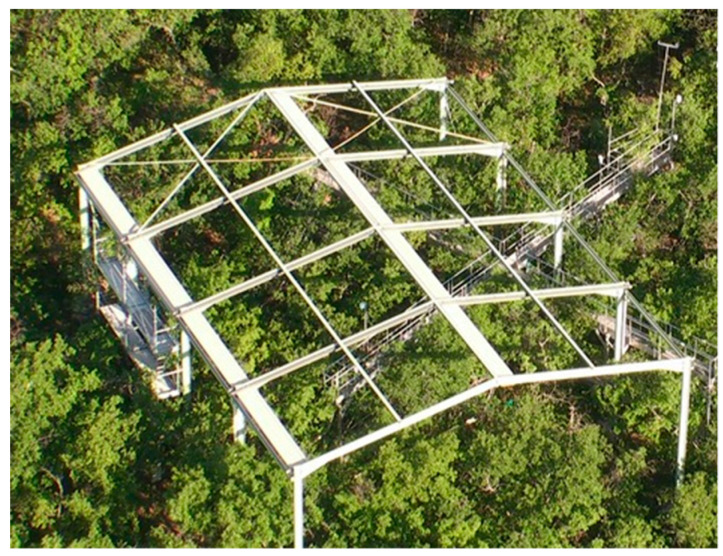
Oak Observatory of the Observatoire de Haute-Provence (O_3_HP). 43°56′06.74″ N, 5°42′38.57″ E. Rain exclusion device and the scaffolding for canopy measurements.

Leaf samples were collected at the Oak Observatory of the Observatoire de Haute Provence (O_3_HP) in the vicinity of Manosque in Southern France (43°56′06.74″ N, 5°42′38.57″ E). The O_3_HP site is situated in a 100-year old forest dominated by Downy oak (*Quercus pubescens*) mixed with Montpellier maple (*Acer monspessulanum,* 25% of leaf biomass). Since 2012, a rain exclusion device consisting of a retractable roof was built over a plot of 300 m^2^, thus allowing us to apply a precipitation scenario close to a reduction in annual rainfall of about 30–40%. The control plot (300 m^2^) is close to the exclusion plot. The upper and lower canopy is accessed by a scaffolding (Figure 1). Five mature leaves per tree were harvested at the upper canopy level at solar noon, then pooled and immediately frozen in liquid nitrogen and stored at −80° until used. Ten trees were selected for each plot and leaf collection was carried out in spring (26 May 2016) and summer (8 September 2016).

### 2.2. RNA Extraction

Total RNA was isolated using the RNeasy Plant Mini Kit (Qiagen, France SAS, Villebon-sur-Yvette, France) following the manufacturer’s instructions with minor modifications. The extracted RNA samples were then qualitatively and quantitatively checked using the Agilent 2100 Bioanalyzer with an RNA Nano chip and stored at −80 °C until further analyses. Ten replicates per plot and date were realized, resulting in forty samples extraction.

### 2.3. Library Preparation and Sequencing

The cDNA library for RNA-seq was constructed using TruSeq™ RNA Kit (Illumina, San Diego, CA, USA) according to the manufacturer’s instructions, and was then sequenced using Next Seq (Illumina Inc., San Diego, CA, USA) to obtain ∼150 bp sequences from both ends of each cDNA. Poly-A selection was applied and two runs were carried out, which generated 50 million reads per sample in paired-end sequencing.

### 2.4. Transcriptome Assembly and Completeness

After quality assessment, adaptors were trimmed and low-quality reads (average quality control QC < 30) were filtered with Trimmomatic v0.38 [14] raw reads, which were then used for several transcriptome assembly tests using Trinity [15], CLC-assembly-cell-4.1.0, BinPacker [16]. Reads were also remapped (HISAT2-2.0.5) [17] against the genome of the close species *Quercus robur* available in the European Nucleotide Archive (ENA) (PRJEB7855). Mapping results were used to build a genome-guided de novo transcriptome assembly using the Trinity assembler (v2.5.1). Finally, only transcripts with at least a Transcripts Per Million (TPM) value above 1 and isoforms corresponding to more than 1% of the total gene count were kept. Transcriptome assembly completeness was evaluated by pseudo-mapping the sequencing reads back to assembly using Kallisto via trinityrnaseq-2.5.1 [18] and BUSCO-3.0.2 analysis [19].

### 2.5. Functional Annotation

Peptide prediction was performed using TransDecoder-3.0.1 [20]. A similarity search (Diamond of the Transdecoder predicted peptides) was performed against the UniProt/SwissProt, the Uniref90 and the nr databases (release: 5 December 2017). Peptide signal prediction was performed using SignalP v4. [21]. Transmembrane peptide detection was performed using TMHMM v2.0c [22]. Protein domain search was performed using hmmscan from the hmmer v.3.1b2 suite against the Pfam-A database [23] version 29.0. Finally, transcriptome functional annotation was performed using the RNAmmer and Trinotate-3.0.2 pipeline [20]. 

### 2.6. Identification of Differentially Expressed Genes Involved in Drought Tolerance and Gene Ontology Enrichment

Differential gene expression between drought treatments was identified using R software [24] and the DESeq2, edgeR and pcaMethods packages. The RNA transcripts with adjusted (Bonferroni correction) *p*-values < 0.001 and absolute fold changes ≥ 4 were considered as differentially expressed. A GO (gene ontology) term enrichment analysis was conducted jointly using the goseq R package. All these analyses were conducted using companion scripts of the Trinity package. To visualize the interactions between the differentially expressed gene datasets, Venn diagrams were realized in R with the Vennerable package. 

### 2.7. Targeted Metabolomics by Gas Chromatography/Mass Spectrometry (GC/MS) Analysis

Extraction: Sample material (50 mg) was extracted in 360 µL of 100% MeOH for 15 min at 70 °C with vigorous shaking. After the addition of 200 µL of chloroform, samples were shaken at 37 °C for 5 min. To separate polar and organic phases, 400 µL of water was added, and samples were centrifuged for 10 min at 11,000× *g*. As internal standard, 20 µL of ribitol (0.2 mg/mL) was added to each sample. For the derivatization, 300 µL of the polar (upper) phase was transferred to a fresh tube and dried in a vacuum concentrator (speed-vac, Thermo Scientific, Savant, Germany) without heating.

Derivatization (Methoximation and Silylation): Pellets were re-dissolved in 20 µL of methoximation reagent containing 20 mg/mL of methoxyamine hydrochloride (Sigma 226904) in pyridine (Sigma 270970) and incubated for 2 h at 37 °C with shaking. For silylation, 32.2 µL of N-methyl-N-(trimethylsilyl)trifluoroacetamide (MSTFA; Sigma M7891) and 2.8 µL of alkane standard mixture (50mg/mL C_10_–C_40_; Fluka 68281) were added to each sample. After incubation for 30 min at 37 °C, samples were transferred to glass vials for GC/MS analysis.

Gas Chromatography/Mass Spectrometry (GC/MS) Analysis: A GC/MS-QP2010 Plus (Shimadzu^®^, Shimadzu Corporation, Kyoto, Japan) fitted with a Zebron ZB 5MS column (Phenomenex^®^; Torrance, CA, USA, 30 m × 0.25 mm × 0.25 µm) was used for GC/MS analysis. A sample of 1 µL was injected at 230 °C in split mode (1:10). The program started with a 1 min hold at 70 °C, followed by a 6 °C/min ramp to 310 °C, a 20 °C/min ramp to 330 °C and a bake-out for 5 min at 330 °C using helium as carrier gas with constant linear velocity. The MS was operated with an ion source, an interface temperatures of 250 °C, a solvent cut time of 7 min and a scan range (m/z) of 40–700 with an event time of 0.2 s.

The “GCMS solution” software (Shimadzu^®^) and the NIST11 Mass Spectral library were used for data processing. Five replicates were carried out for each plot (control and exclusion) in spring and summer. The differential metabolites data were analyzed with MetaboAnalyst 4.0 [25].

### 2.8. Data Accessibility

All sequencing data have been deposited in the NCBI Sequence Read Archive under the accession code PRJNA381515.

## 3. Results

### 3.1. High Throughput Sequencing Output and De Novo Transcriptome Assembly

**Figure 2:**
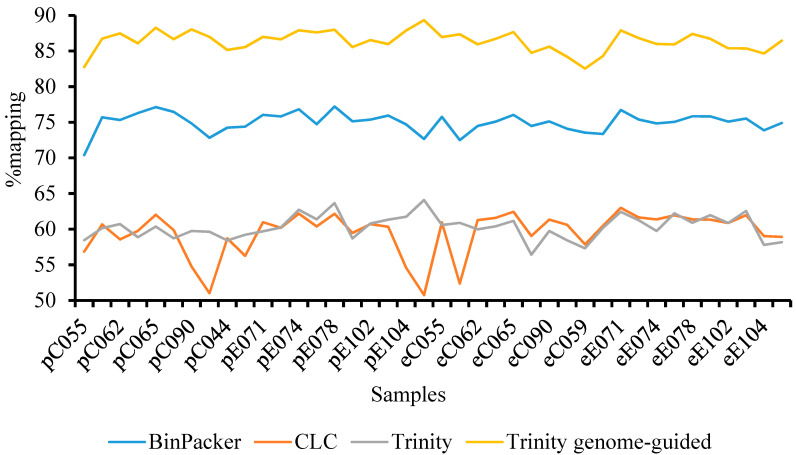
Comparison of pseudoalignment rates between different assemblies. Kallisto analysis with the FR (Forward/Reverse) strand-specific paired-end pseudoalignment option. *Quercus pubescens* leaf samples from control (C) and exclusion (E) plots harvested in spring (p) and summer (e).

Leaves from twenty downy oak trees were used to generate 1.97 Md of paired-end raw reads 150 bp long. After cleaning in order to remove adaptors, low quality and short sequences, this results in 1.79 Md reads representing about 2.5 Tb of data (Table 1). The clean reads were mapped over the previously published de novo transcriptome of *Q. pubescens* [11], but only a covering of 40% was obtained. The studies were thus conducted for de novo transcriptome assembly with different tools, such as BinPacker, CLC and Trinity. The results showed that the read covering rate was also low, ranging from 50 to 75%. This led to a genome-guided transcriptome assembly based on Trinity algorithms using *Quercus robur* contigs as a reference [26]. A high-quality mapping of the clean reads was obtained ranging from 80 to 90% depending on the tested samples (Figure 2). This genome-guided transcriptome assembled more sequences compared to the other assemblers, precisely 530,080 transcripts with a mean length of 595 bp (Table 1). N50 and E90N50 were 780 and 1544, respectively. The filtered version of the assembly (transcripts with TPM > 1) contains 156,986 transcripts. Ninety percent of the global expression is present in 34,572 transcripts. The completeness of the assembly in terms of expected genes was checked through the BUSCO algorithm. As shown in Table 2, among the 303 and 1444 orthologous gene sets of Eukaryota and Embryophyta respectively searched, 92.4% and 83.8% were complete BUSCO single copies or duplicated for Eukaryota and Embryophyta, respectively.

### 3.2. Functional Annotation of the Assembled Contigs

**Figure 3:**
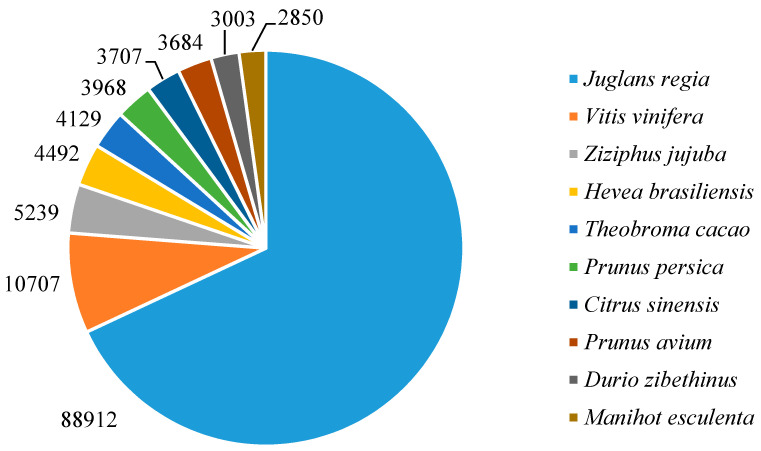
Plant species distribution of DiamondX searches on NCBI nr database of *Q. pubescens* transcripts. Percentage of total top ten homologies. Filtering at E value threshold < 10 × 10^−10^.

Two annotation strategies were carried out: similarity search based on sequence- or functional domain-alignments. As shown in Table 3, there were in the raw transcriptome 200,726 unigenes that exhibited homology to known protein sequences in the Uniref90 BLASTX database (38%) followed by SwissProt BLASTX (27%) and Prot ID (26%) databases. A total of 75,052 contigs (14%) were mapped to the PFAM protein domain/family. For the annotation queries, the default filtering E value was set at <1 × 10^−5^. Sequence homology searches were carried out on the Viridiplantae database, which allows us to identify the species closest to *Q. pubescens*. In order of decreasing homology: *Juglans regia* (88,912 hits) followed by *Vitis vinifera* (10,707 hits), *Ziziphus jujube* (5239 hits), *Hevea brasiliensis* (4492 hits) and *Theobroma cacao* (4129 hits) were the closest species (Figure 3). These results highlight the genome specificity of *Q. pubescens* since, except for the genus *Quercus,* it shares only 17% of the genes expressed with the nearest plant species, namely, walnut.

### 3.3. Differentially Expressed Genes (DEG) Analysis

**Figure 4:**
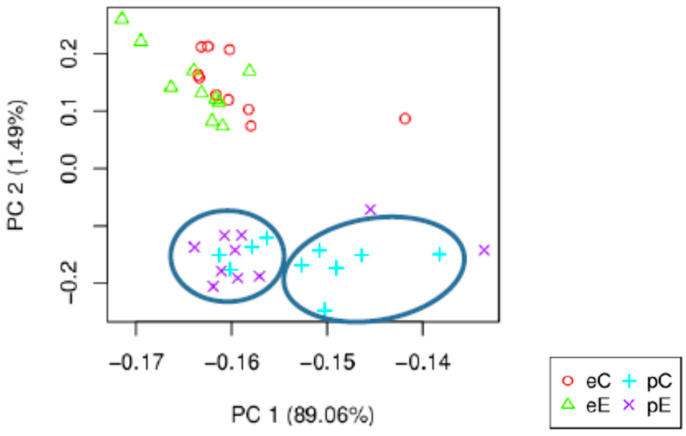
Principal component analysis (PCA) of foliar transcript profile expression of *Q. pubescens* trees; e and p denote sampling in summer and spring, respectively; C and E denote control and rain exclusion treatments, respectively. Circles denote a possible exclusion effect in spring.

**Figure 5:**
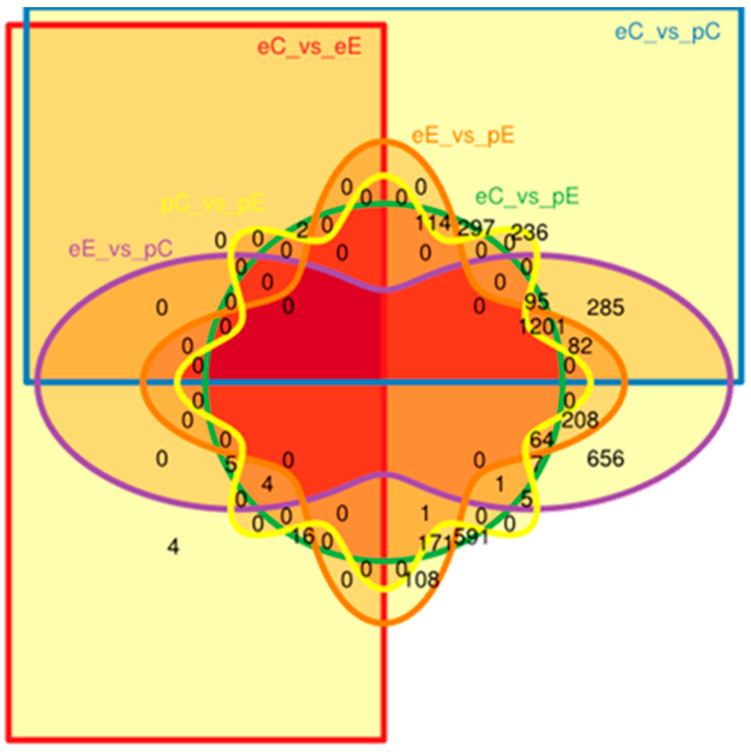
Venn diagram of edegR data showing the intersections between the differentially expressed genes from *Q. pubescens*: eC_vs._eE; eE_vs._pE; eC_vs._pC; pC_vs._pE; eE_vs._pC and eC_vs._pC. Control plot in summer, eC; rain exclusion plot in summer, eE; control plot in spring pC and rain exclusion plot in spring, pE.

In order to address the question of the identification of the genes involved in Downy oak’s response to the reduced precipitation treatment, two strategies were used: the principal component analysis (PCA) and an analysis based on a model using the negative binomial distribution for the estimation of the differential expression of genes (R, packages DESeq2 and edgeR). The PCA representation of the individuals on the first factorial plane 1–2 is displayed in Figure 4. The first two components represent 90% of the total variance with 89 and 1.5% for axes 1 and 2, respectively. Axis 2 clearly exhibited a season effect in terms of gene expression profile, while a slight trend was shown regarding to rain exclusion impact when considering axis 1. Furthermore, the Pearson correlation matrix showed that the correlation coefficient between samples was very high (up to 0.85); most of the expression data are similar and no differences were found. Some samples in summer clearly exhibited a rain exclusion effect. The use of R packages results in the identification of up to 5724 genes. Among these, 42 (31 and 11) were associated with the exclusion treatment, while 4261 were related to the season based on edgeR algorithms (Table 4). Further analysis showed that DESeq2 and edgeR share 3828 features. The Venn diagram, based on EdgeR differential analysis data, identified transcripts specific or common to different modalities (Figure 5). In particular, four of the 4153 differentially expressed genes are specific to the summer drought (eC vs. eE). The effect of seasonal variation (eC vs. pC) is determined by 236 transcripts, while the combined effect of treatment and season (eE vs. pE) is regulated by 108 features. We will focus on the 42 differentially expressed genes due to the aridification of the climate which exhibited 4 common features between spring and summer (Figure S2). When available, the corresponding TAIR (The *Arabidopsis* Information Resource) gene ID of *Q. pubescens* differential genes or contigs was provided (Table S1).

**Figure 6:**
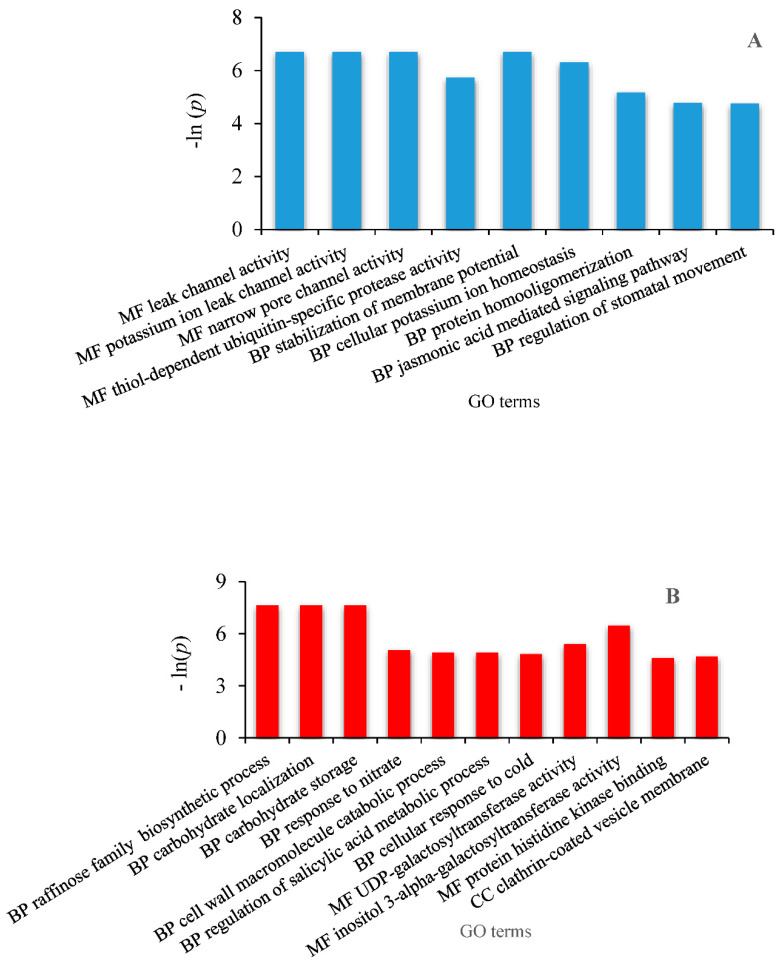
GO annotation of differential transcripts of *Q. pubescens* in spring as a function of the enrichment *p* value *p* < 0.01. (**A**) Upregulated features and (**B**) downregulated features. MF, molecular function; BP, biological process; CC, cellular component.

**Figure 7:**
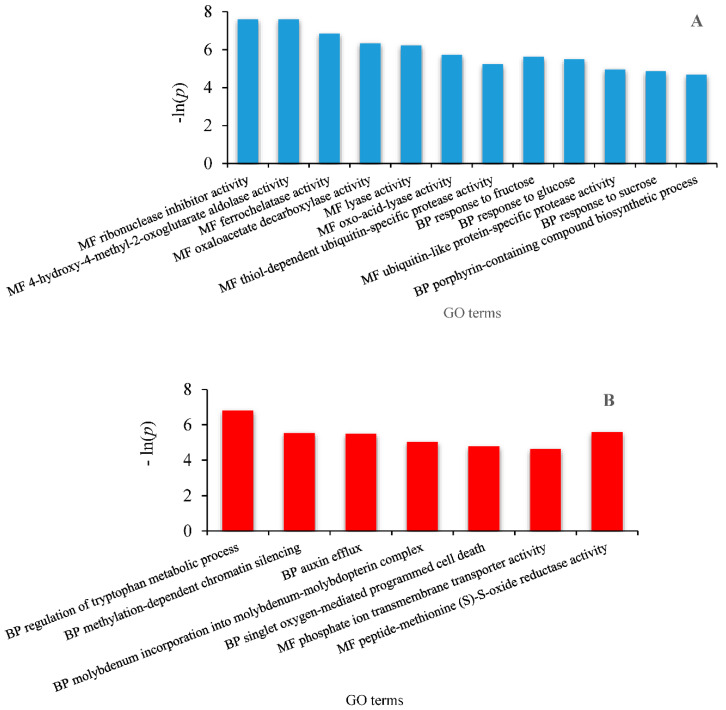
GO annotation of differential transcripts of *Q. pubescens* in summer as a function of the enrichment *p* value *p* < 0.01. (**A**) Upregulated features and (**B**) downregulated features. MF, molecular function; BP, biological process.

The GO (Gene Ontology) terms of the 42 differential expressed transcripts obtained from the edgeR algorithm are presented in Figure 6 and Figure 7 which reflect the effect of climate aridification according to the seasons.

**Spring DEG. **Soil moisture levels at the sampling date were about 25% and 20% in controls and rain exclusion, respectively (Figure S1). Differential transcripts were nevertheless expressed. Upregulated features concern GO categories related to molecular function and biological processes (Figure 6A). The main molecular function that is exacerbated because of aridity is the activity of cation channels, precisely, that of potassium leak channels. This results in stomatal closure mediated by the jasmonic acid signal transduction pathway.

Downregulated genes are mainly related to biological processes, molecular function and cellular components of GO categories (Figure 6B). The most downregulated features involve the biosynthesis of raffinose oligosaccharides (RFOs) through the activity of the inositol 3-α-galactosyltransferase.

**Summer DEG.** Soil moisture levels in controls and rain exclusion were 13% and 11%, respectively (Figure S1). Upregulated genes concern GO categories that are related to molecular function and biological process (Figure 7A). Among the molecular functions identified, lyase activity was upregulated mainly by that of 4-hydroxy-4-methyl-2-oxoglutarate aldolase. The enzyme catalyzes the breakdown of 4-hydroxy-4-methyl-2-oxoglutarate (HMG) into two molecules of pyruvate. Furthermore, it contains a secondary oxaloacetate (OAA) decarboxylase activity because of the common pyruvate enolate intermediate formed following the breakdown of the C-C bond in the retro-aldolization and decarboxylation reactions. HMG aldolase also exhibited putative ribonuclease inhibitor activity. Among other molecular functions, ferrochelatase was identified. Additionally, the thiol-dependent ubiquitin-specific protease activity was enhanced as in spring. Ubiquitin-specific proteases (UBPs) are a family of hydrolases that remove polypeptides covalently linked to the C-terminal glycine of ubiquitin. UBPs regulate the ubiquitin/26S proteasome proteolytic pathway through deubiquitination, thus recycling ubiquitins during the breakdown of ubiquitin–protein conjugates. In turn, the main biological processes deal with the response to sucrose and monosaccharides and also with heme metabolic pathways including porphyrin-containing compounds biosynthesis.

Of the 12 genes downregulated in summer, only two belong to the category of molecular functions—the rest being involved in biological processes (Figure 7B). Peptide methionine (S)-S-oxide reductase A (PMR-A) catalyzes the reduction of oxidized methionine (S) in proteins/peptides. Another protein whose activity was downregulated is a phosphate ion transmembrane transporter. The biological processes affected by drought in summer concern hormonal, amino acid, mineral, DNA and oxidative metabolism. For plant survival, the most important molybdenum enzyme is the cytosolic nitrate reductase, which catalyzes the first step of nitrate assimilation. A weak activity of this enzyme was shown upon drought as well as a modification of chromatin structure.

### 3.4. Targeted Metabolomic

**Figure 8:**
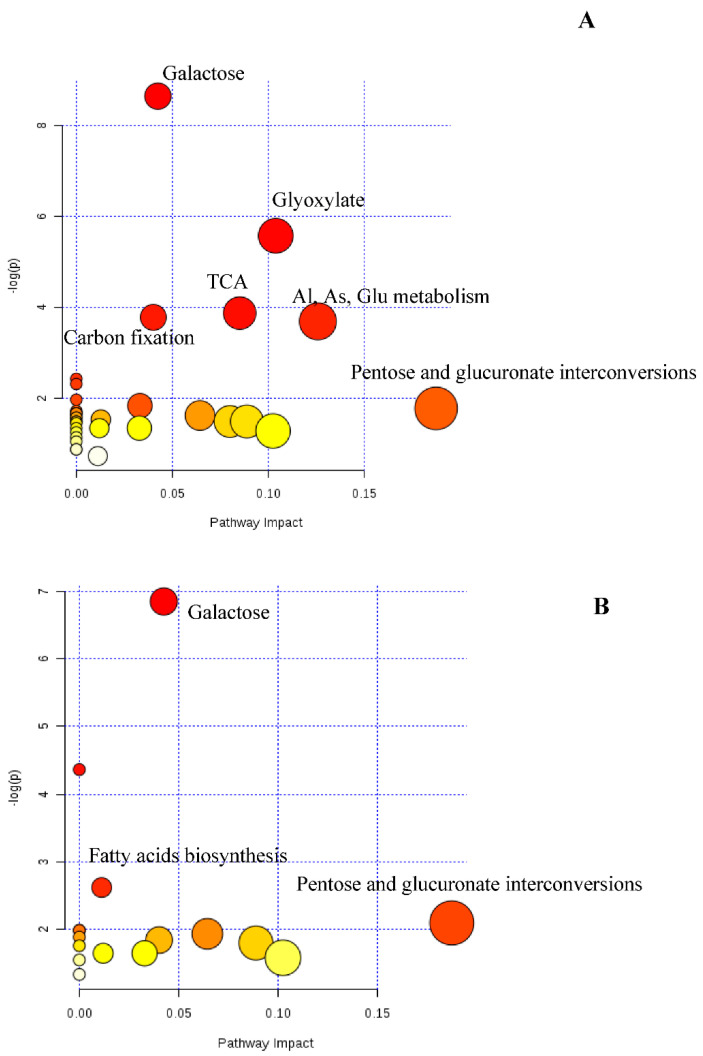
Map of enriched metabolic pathways from differential metabolites of *Quercus pubescens* leaves; rain exclusion versus control during spring (**A**) and summer (**B**). Topological analysis of impact factors and the *p* value by pathway enrichment analysis. The color intensity (white to red) indicates the increasing *p* value, while the circle diameter covaries with pathway impact.

Twenty-three metabolites were identified in leaf samples (Table 5). As in RNA profiling, several metabolites were drought- and/or season-specific. Spring rain exclusion promoted more than a 1.5-fold increase in lactic acid, pyroglutamic acid, xylulose, isocitric acid and gallic acid contents in leaves of the exclusion plot as compared to control. However, a decrease in the accumulation and/or biosynthesis of myo-inositol, quinic acid and shikimic acid was observed. In summer, very few metabolites were differentially accumulated in the drought condition, namely, oxalic acid (2-fold), pyroglutamic acid (2-fold) and malic acid (1.6-fold). It should be noted that both seasons share the same differential increase in pyroglutamic acid and also the decrease in catechin (0.5-fold) contents in terms of adaptation to water stress. When considering the effect of the season, xylulose and sorbitol were the main sugar and sugar alcohol markers. Through metabolic pathway enrichment, it appears that the response of *Q. pubescens* to drought involves principally galactose, glyoxylate, a tricarboxylic acid (TCA) cycle and amino acid metabolisms in spring (Figure 8A).

## 4. Discussion

Our study provided the first genome-guided transcriptome of *Q. pubescens* leaves. Samples originate from individuals that were grown in situ on a site that has been a forest since the 18th century. As such, this dataset may be considered as a fundamental reference. Although high-throughput sequencing now allows one to assemble transcriptomes for non-model species, our comparative study has shown that assembly errors such as the formation of partial or chimeric transcripts can deeply affect the quality of the assemblies, and we demonstrate the utility of using closely related species to perform genome-guided de novo transcriptome assembly [28]. A sequence homology search revealed that *Q. pubescens* exhibited the highest similarity with *J. regia* (Figure 3).

Differential analysis allowed us to identify four key genes common to both spring and summer, of which three are annotated: upregulated ubiquitin carboxyl-terminal hydrolase 12-like; downregulated putative calcium-binding protein cml26; and nifU-like protein 1, chloroplastic (Figure S2).

In a previous study carried out on the site of O_3_HP, a two-fold increase in stomata closure was found in spring from *Q. pubescens* of the rain exclusion treatment as compared to the control plot [29].

Among spring DEG, an ionic channel gene was upregulated (Figure 6A). Several reports have shown that the increase in K^+^ efflux in stomata cell guards results in stomata closure [30,31,32]. This closure is mediated by both abscisic acid (ABA)-dependent and -independent signaling pathways [33]. Drought also induces the occurrence of ROS (Reactive Oxygen Species), which in turn mediate abscisic acid (ABA)- and jasmonate-induced stomatal closures [34,35]. It appears that drought induces *Q. pubescens* stomata closure in the signaling network of both ABA and jasmonic acid.

RFO transcripts were downregulated (Figure 6B); they are described as the most abundant galactose containing oligosaccharides in the plant kingdom. They are α-galactosyl extensions of sucrose, whose predominant functions are related to transport and storage [36]. While RFOs were identified as differential genes from plants submitted to abiotic and biotic stresses, their role remains unclear [36]. In our study, the downregulation of the biosynthesis of RFOs and more generally galactose metabolism may be interpreted as the need of carbon resource as a consequence of stomatal closure during spring.

Concerning summer DEG, despite the slight difference in soil moisture levels in control compared to rain exclusion (Figure S1), differential transcripts were expressed. Because the exclusion device was set up since 2010, it is likely that the response obtained is rather the consequence of long-term drought application. One may consider that the metabolic activity of *Q. pubescens* in response to the summer drought is pyruvate rate-limiting. Since the substrate of HMG aldolase is also an oxaloacetate, an intermediate of the tricarboxylic acid cycle, it is likely that drought stimulates its accumulation through the pyruvate carboxylation by the anaplerotic pathway. Another key upregulated transcript is ferrochelatase, whose sequence exhibited a high similarity with *Quercus suber* chloroplastic ferrochelatase-2 (FC2). The enzyme has been functionally characterized as involved in the biosynthesis of the heme of photosynthetic cytochromes and stress response proteins [37]. Our data are in accordance with previous studies that showed that stress induced by salinity and water stress result in upregulated genes of ferrochelatase [38,39,40].

UBPs helps confer *Arabidopsis thaliana* resistance to abiotic stress [41], and the upregulated level of the enzyme found in our study may be interpreted as the need for metabolic control by increased protein turnover during water stress. Peptide-methionine (S)-S-oxide reductase A (PMR-A) was shown to enhance cell resistance to ROS (reactive oxygen species) and to be located in tobacco chloroplasts [42]. An increase in resistance to drought and plant-pathogen attacks was also proposed upon identification of the overexpression of the PMR gene [43,44,45]. Since methionine is known as the most sensitive amino acid to oxidation in proteins or peptides, the downregulation of PMR-A in *Q. pubescens* may be interpreted as an accelerated proteolytic activity in view of the fact that specific amino acids demand satisfaction.

Summer stomatal conductance was significantly reduced to 56% in control as compared to the rain exclusion plot with a concomitant reduction of net photosynthesis [29]. Hence, it is probable that the decrease in phosphate ion transmembrane transporter activity reflects a balance of the redox state between photosynthesis light reactions and the Calvin cycle. Similarly, a decrease in the respiratory intensity could be considered, thus limiting the risk of generating ROS due to the importance of the electronic transport flow. The plastic growth and adaptability of plants to the external environmental conditions are mainly mediated by hormones of which auxin plays a fundamental role. Its biological function is determined by the strict coordination of three complex processes: auxin metabolism, auxin transport, and auxin signaling [46]. The downregulation of tryptophan, the precursor of auxin biosynthesis, as well as that of auxin efflux, suggest that drought impairs auxin biosynthesis and transport. Our results seem to be in contradiction with other works that show that the application of auxin improves plant resistance to water stress [47,48]. Indeed, auxin efflux is mediated by specific PIN-FORMED transporters regulating the asymmetric spatiotemporal distribution of auxin in the whole plant. The downregulation of auxin efflux may result in a high accumulation of auxin more than that would be expected from the tryptophan biosynthetic pathway. The weak activity of molybdenum incorporation into the molybdenum–molybdopterin complex may be explained by a reduction in ATP (Adenosine Triphosphate) input from photosynthetic light reactions at the expense of nitrification during drought. Light absorbed in excess could induce a light-dependent cell death, a form of programmed cell death (PCD) that is unique to plants. The requirement of PCD has often been associated with the production of reactive oxygen species during photosynthesis [49]. Hence, the mitigated singlet oxygen-mediated PCD likely upstream of jasmonate would reduce the deleterious effects of ROS in downy oak during water stress. In many plant species, drought was accompanied by DNA methylation [50]. The downregulation of methylation-dependent chromatin silencing is in accordance with the need for the recruitment of specific transcripts in response to aridification.

Differential gene analysis in more severe drought conditions reveals that several features were involved in the tricarboxylic acids cycle and carbohydrate and amino acid metabolisms. This motivated us to conduct a directed metabolome analysis accordingly.

The large accumulation of malate and isocitrate (Table 5) may be interpreted as a tricarboxylic acid (TCA) shunt in favor of the glyoxylate cycle. Indeed, the glyoxylate cycle is an alternative to the production of glucose from AcetylCoA given the low availability of CO_2_. Water stress experiments in rice demonstrated a high expression of genes encoding isocitrate lyase and malate synthase, two key enzymes of the glyoxylate cycle [51]. In addition, there is an increase in oxalic acid concentrations, an intermediate product of the glyoxylate cycle but also recognized as being an environmental stress marker [52]. The metabolic profiling thus produced shows similarities with the gene expression pattern. Indeed, during the summer, the increased expression of many genes confirms a switch in the energetic metabolism towards the cyclic pathway of glyoxylate and that of gluconeogenesis to the detriment of the TCA cycle. In particular, the upregulation of the genes encoding oxaloacetate decarboxylase causes a high accumulation of pyruvate, and that of the oxo-acid lyase genes is correlated with the increased production of isocitrate through isocitrate lyase activity. This latter activity is supported by the strong accumulation of malate from the reaction between AcetylCoA and glyoxylate catalyzed by malate synthase. Furthermore, the downregulation of the inositol 3-α-galactosyltransferase activity is correlated with the decrease in myo-inositol content in response to drought. Throughout the seasons, drought induces an increasing accumulation of pyroglutamic acid (Table 5). This result is in accordance with the finding that the application of pyroglutamic acid highly promotes water stress tolerance effects in deficit irrigated lettuce, probably as a precursor of proline biosynthesis [53]. However, a cyclization of free glutamine and glutamate to free pyroglutamic acid in electrospray ionization-based mass spectrometry is not excluded [54].

**Figure 9:**
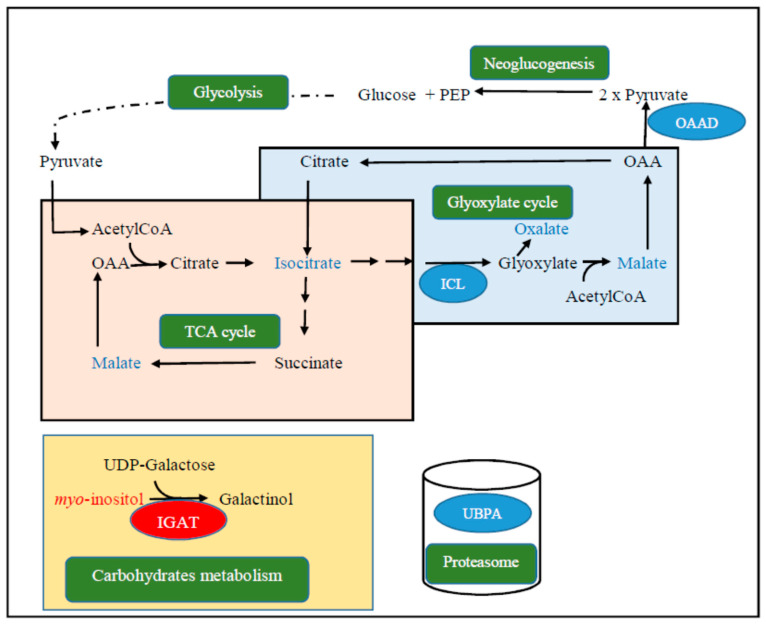
Hypothetical scheme of the early metabolic pathways involved in the adaptation of *Quercus pubescens* to drought. Complex proteins and metabolic pathways (green); proteins of upregulated transcripts (blue circle); upregulated metabolites (blue); downregulated metabolite (red); protein of downregulated transcript (red circle). Oxalocetate (OAA); oxaloacetate decarboxylase (OAAD); ubiquitin-like-protein-specific protease activity (UBPA); inositol 3-α-galactosyltransferase activity (IGAT); phosphoenolpyruvate (PEP); uridin diphosphate (UDP); tricarboxylic acid (TCA); isocitrate lyase (ICL); coenzymeA (CoA).

Of the differentially accumulated metabolites and transcripts, we thus proposed a hypothetical scheme of the early response of *Q. pubescens* to in situ water withholding conditions (Figure 9). One of the consequences of stomata closure is the imbalance in the redox state between light and dark reactions of photosynthesis. To promote the availability of carbohydrates, we hypothesized a shunt of the TCA cycle through the glyoxylate cycle in relation to the formation of oxaloacetate. The latter compound is decarboxylated by an oxaloacetate decarboxylase, leading to the formation of two molecules of pyruvate that enter either into the neoglucogenesis pathway or as a precursor of AcetylCoA formation. Simultaneously, the carbohydrate storage rate is reduced since the activity of the inositol 3-α-galactosyltransferase is downregulated. Furthermore, the upregulation of ubiquitin-like-protein-specific protease activity suggests that drought favors intense proteolytic activities coupled with the availability of free ubiquitin.

In conclusion, the present work provides an important dataset on downy oak genomics. We assembled for the first time the genome-guided transcriptome of *Q. pubescens*. This work generated 1.7 Md of paired-end long reads of 150 nucleotides, therefore contributing to feed the genomic database of the genus *Quercus* hitherto limited to a few species. Potential functions of about 102,000 filtered transcripts from *Q. pubescens* were described in this study. The application of long-term water stress (four years) at O_3_HP site triggers adaptive processes, which confirms the expected genomic plasticity in regard to global changes. The differential analysis of transcripts coupled with the targeted metabolomics allowed us to demonstrate the overlap of changes in gene expression with the occurrence of some metabolites, particularly in relation to the TCA cycle. That said, the Krebs cycle might be involved in the early metabolic changes in plant response to drought together with the glyoxylate cycle for de novo synthesis of glucose. The highly upregulated gene of ubiquitin carboxyl-terminal hydrolase 12-like plays an important role in ubiquitin recycling during protein degradation. Other candidate genes were identified, mainly those of ferrochelatase 2 and porphyrin metabolism involved in photosynthesis electron transport; galactose metabolism and carbohydrate storage; and antioxidative defense by downregulating singlet oxygen-mediated PCD. However, the initiating events that trigger the regulation of these genes are unclear, although an epigenetic mechanism related to the heterochromatic gene silencing was shown. Further studies of functional genomics will be necessary to confirm or invalidate the role of the different identified candidate genes. Specifically, this study, while aiming to elucidate the metabolic pathways involved in plants tolerance to drought, can also serve as a resource for further functional genomic and phylogenetic analyses on the group of species of the *Quercus* genus.

## Figures and Tables

**Table 1:** Summary of RNA-Seq and genome-guided sequence assemblies (raw and filtered) of *Q. pubescens.* TPM (Transcripts Per Kilobase Million).

Total raw paired-end reads	1,971,431,570 × 2
Total clean paired-end reads	1,794,615,817 × 2
Number of transcripts/trinity “genes”—raw assembly	530,080/395,969
Mean contig length (bp)/N50—raw assembly	595/780
E90N50/E90 number of transcripts	1544/34,572
Number of transcripts/trinity “genes”—filtered transcriptome (TPM < 1)	156,986/126,106
Mean contig length (bp)/N50—filtered transcriptome (TPM < 1)	720/1133

**Table 2:** BUSCO run for genome-guided de novo transcriptome assembly of *Q. pubescens* and comparison with *Q. robur* de novo assembly (OCV4_assembly_final.fas) [27].

Transcriptomes Assemblies	*Q. pubescens* Filtered	*Q. pubescens* Raw	*Q. robur*	*Q. pubescens* Filtered	*Q. pubescens* Raw	*Q. robur*
BUSCO sets	Eukaryota			Embryophyta		
Complete BUSCOs (C)	280	288	140	1206	1266	554
Complete BUSCOs%	92.40	95.10	46.30	83.80	87.90	38.40
Complete and single-copy BUSCOs (S)	112	103	115	645	601	447
Complete and duplicated BUSCOs (D)	168	185	25	561	665	107
Fragmented BUSCOs (F)	14	12	20	105	113	104
Missing BUSCOs (M)	9	3	143	129	61	782
Total BUSCO groups searched	303	303	303	1440	1440	1440

**Table 3:** Summary of annotations of raw and filtered *Q. pubescens* genome-guided sequence assembly. KEGG (Kyoto Encyclopedia of Genes and Genomes); eggNOG database (evolutionary genealogy of genes: Non-supervised Orthologous Groups); PFAM (Protein family); TMHMM Transmembrane Helices Hidden Markov Models; RNAmmer (consistent and rapid annotation of ribosomal *RNA* genes).

Database	*Q. pubescens* Raw		*Q. pubescens* Filtered	
Number of transcripts	530,080		156,986	
	Number of transcripts/peptides annotated	Percentage of transcripts/peptides annotated	Number of transcripts/peptides annotated	Percentage of transcripts/peptides annotated
Uniref90 DiamondX	214,856	40.54	102,445	65.26
SwissProt DiamondX	155,684	29.37	79,367	50.56
GO Extract from Sequence Similarity Search	146,689		75,852	
Number of Protein Predicted	135,957		71,166	
KEGG	124,285	91.14	67,348	94.63
Uniref90 DiamondP	120,789	88.85	66,374	93.27
EggNOG Function	118,026	86.81	63,724	89.54
SwissProt DiamondP	95,098	69.94	54,967	77.24
PFAM	86,021	63.27	49,462	69.5
GO extract from PFAM Analysis	52,107		31,994	
TmHMM	22,131		11,986	
RNAMMER	110		68	

**Table 4:** Differentially expressed genes (DEG) of *Q. pubescens* leaves in response to the sampling season and the rain exclusion treatment. The thresholds of statistical tests were fixed at p_adj_ < 10^−3^ and log2FC ≥ 2. 5724, and 4153 DEG were detected by DESeq2 (bold) and edgeR, respectively; e and p denote sampling in summer and spring, respectively; C and E denote control and rain exclusion treatments, respectively.

Samples	eC	eE	pC	pE
eC	/	**15**	**3122**	**3529**
eE	31	/	**3460**	**3709**
pC	2312	2613	/	**2**
pE	2569	1949	11	/

**Table 5:** Targeted metabolomics of the trimethylsilyl/ethoxime (TMS) derivatized organic acids and sugars from leaf extracts of *Quercus pubescens*. * According to the NIST (National Institute of Standards and Technology) database. Data of the ratios are means of five independent extractions. E, rain exclusion; C, control; n.d., no data. No change is considered for a fold change of one.

Name	Fold Change			
	Summer	Spring	Spring/Summer	
	E/C	E/C	C	E
Lactic acid	1.0	1.6	0.7	0.4
Oxalic acid	1.8	1.2	0.4	0.6
Phosphoric acid	1.2	n.d.	n.d.	0.8
Glycerol	1.0	1.3	0.9	0.7
Succinic acid	1.0	1.2	0.4	0.4
Aspartic acid	n.d.	1.1	n.d.	0.6
Pyroglutamic acid	1.8	2.1	1.3	1.1
Xylulose	0.9	1.8	9.8	4.9
Isocitric acid	1.0	1.6	0.9	0.6
Sorbitol	1.4	1.1	2.1	2.5
Viburnitol *	0.9	1.2	0.9	0.7
Inositol, myo-	1.2	0.6	0.7	1.2
Sucrose	1.1	1.1	0.6	0.6
Quinic acid	0.8	0.6	0.6	0.8
D-Ketohexose sugars	1.2	0.9	0.8	1.2
D-Aldohexoses	1.0	0.9	1.0	1.1
Erythronic acid	0.8	0.7	0.9	1.0
Hexadecanoic acid	0.8	1.1	1.5	1.2
Octadecanoic acid	0.8	1.0	1.0	0.8
Malic acid	1.6	1.3	1.2	1.5
Shikimic acid	n.d.	0.5	n.d.	n.d.
Gallic acid	1.0	1.6	0.6	0.4
Catechin	0.5	0.5	0.3	0.3

## Supplementary Materials

The following are available online at https://www.mdpi.com/2223-7747/9/9/1149/s1, Figure S1: O_3_HP soil water status taking into account the different sampling dates. Figure S2: Differential transcripts of leaves of *Quercus pubescens* annotated from an NCBI Blastn search: (a) spring and (b) summer. Blue, upregulated features; red, downregulated features. Table S1: *Quercus pubescens* genes or contigs differentially expressed during rain exclusion treatment.
