# Supplementary material for: Response of Downy Oak (Quercus pubescens Willd.) to Climate Change: Transcriptome Assembly, Differential Gene Analysis and Targeted Metabolomics
Source: Plants (Basel). 2020 Sep 4;9(9):1149. doi: 10.3390/plants9091149 (PMC7570186; doi:10.3390/plants9091149)
Supplement: Supplementary file 1 [file plants-09-01149-s001.pdf]

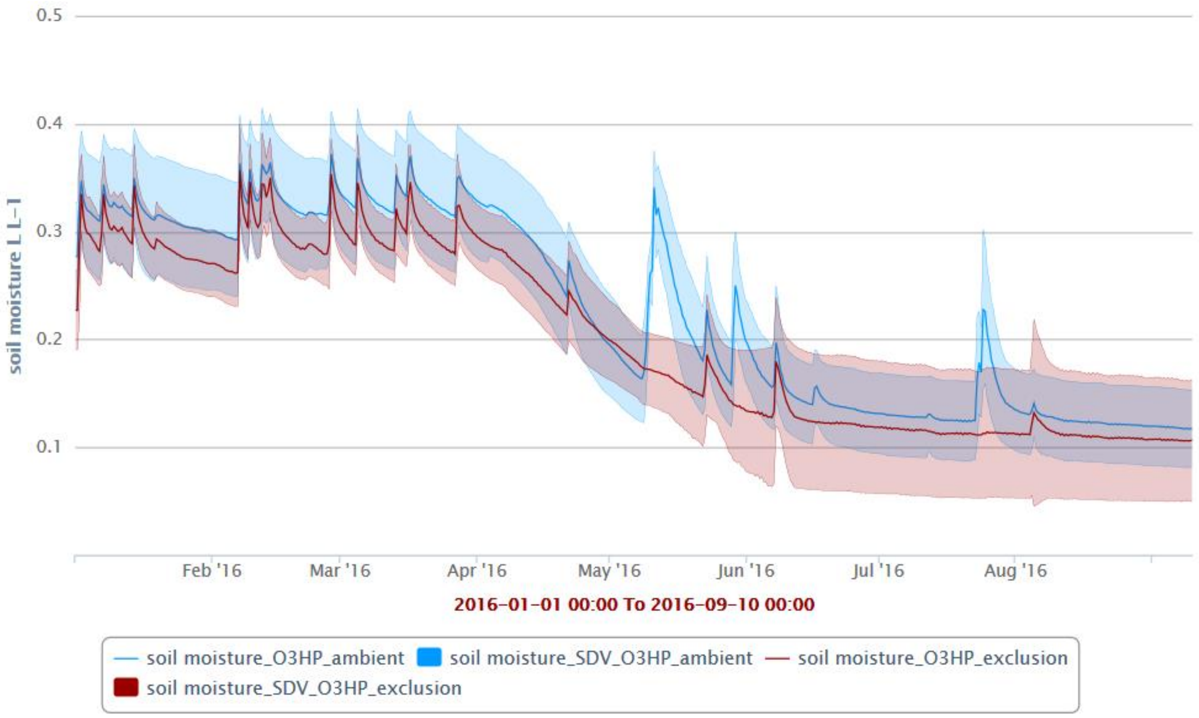

Supplementary fig. S1. O_3HP soil water status taking into account the different sampling dates.

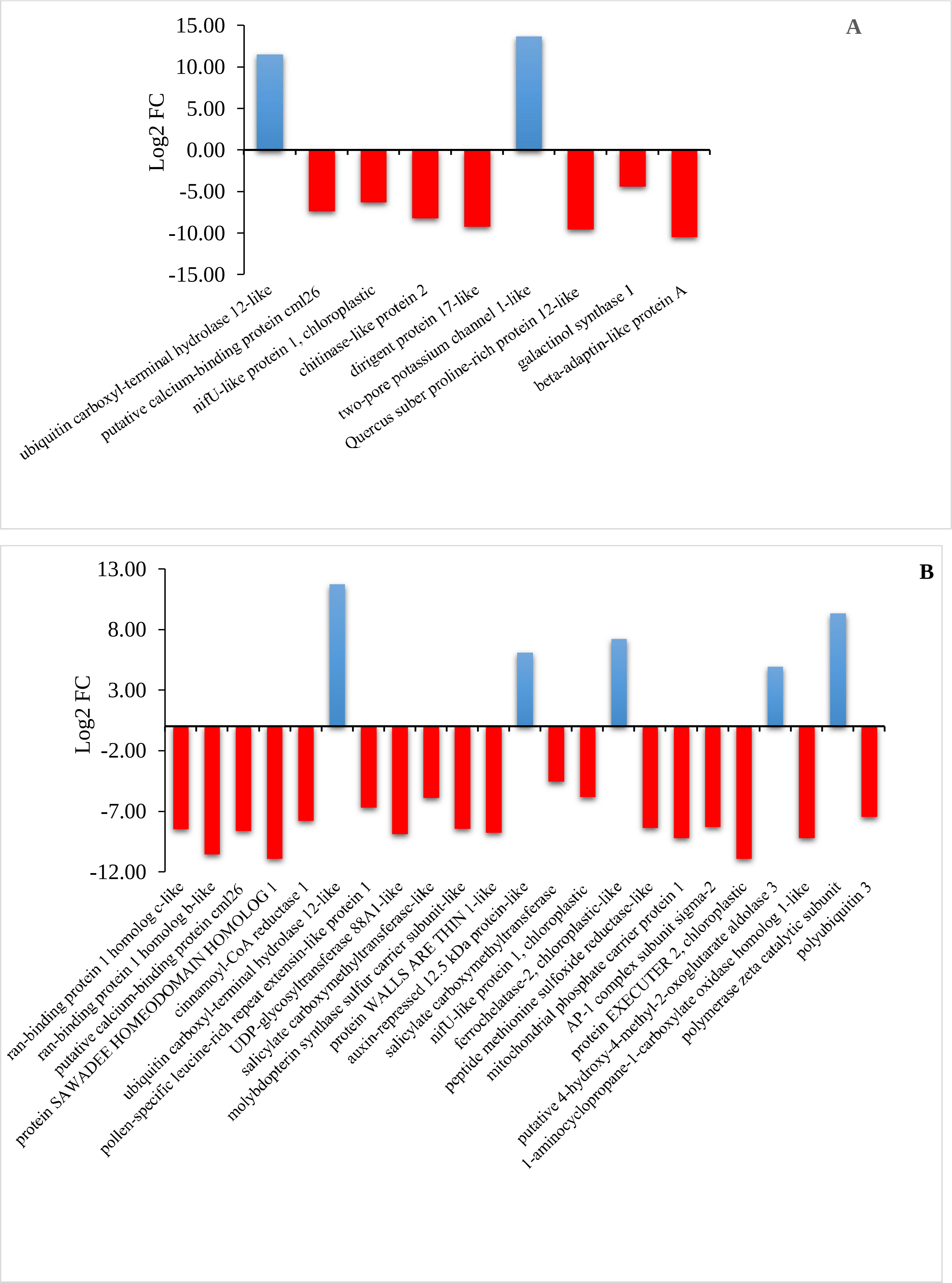

Supplementary fig. S2. Differential transcripts of leaves of *Quercus pubescens* annoted from NCBI Blastn search. (a); spring; (b), summer. Blue up-regulated, red: down-regulated features.

Trinity gene or contig ID	Protein or domain name	TAIR ID	Season
GG_50425_c4_g1_i1	ubiquitin carboxyl-terminal hydrolase 12-like	AT5G06600	Spring
GG_11108_c69_g1_i1	putative calcium-binding protein cml26	AT1G73630	Spring
GG_14487_c10_g1_i1	nifU-like protein 1, chloroplastic	AT4G01940	Spring
GG_44881_c17_g1_i1	chitinase-like protein 2	AT3G16920	Spring
GG_39825_c29_g1_i1	dirigent protein 17-like		Spring
GG_11622_c28_g1_i1	two-pore potassium channel 1-like		Spring
GG_39053_c0_g1_i1	Quercus suber proline-rich protein 12-like		Spring
GG_13230_c62_g1_i1	galactinol synthase 1	AT2G47180	Spring
GG_4958_c0_g1_i1	28S ribosomal RNA gene, complete sequence		Spring
GG_41960_c44_g1_i5	beta-adaptin-like protein A	AT5G11490	Spring
GG_47228_c14_g1_i2	ran-binding protein 1 homolog c-like	AT5G58590	Summer
GG_50049_c0_g1_i1	ran-binding protein 1 homolog b-like	AT2G30060	Summer
GG_11108_c69_g1_i1	putative calcium-binding protein cml26	AT1G73630	Summer
GG_4958_c0_g1_i1	28S ribosomal RNA gene, complete sequence	AT1G80570	Summer
GG_38843_c72_g1_i1	protein SAWADEE HOMEODOMAIN HOMOLOG 1	AT1G15215	Summer
GG_14366_c53_g1_i1	cinnamoyl-CoA reductase 1	AT1G15950	Summer
GG_50425_c4_g1_i1	ubiquitin carboxyl-terminal hydrolase 12-like	AT5G06600	Summer
GG_22151_c4_g1_i1	pollen-specific leucine-rich repeat extensin-like protein 1	AT3G19020	Summer
GG_53011_c4_g1_i1	Unknown		Summer
GG_47645_c29_g1_i2	UDP-glycosyltransferase 88A1-like	AT3G16520	Summer
GG_38151_c33_g1_i1	salicylate carboxymethyltransferase-like		Summer
GG_53011_c4_g1_i1	Unknown		Summer
GG_31096_c0_g1_i1	molybdopterin synthase sulfur carrier subunit-like		Summer
GG_38972_c72_g1_i2	protein WALLS ARE THIN 1-like	AT1G75500	Summer
GG_13447_c40_g1_i2	auxin-repressed 12.5 kDa protein-like		Summer
GG_7481_c106_g1_i3	salicylate carboxymethyltransferase	AT3G11480	Summer
GG_14487_c10_g1_i1	nifU-like protein 1, chloroplastic	AT4G01940	Summer
GG_13694_c66_g1_i4	ferrochelatase-2, chloroplastic-like		Summer
GG_40710_c64_g1_i1	peptide methionine sulfoxide reductase-like		Summer
GG_49255_c27_g1_i2	mitochondrial phosphate carrier protein 1	AT2G17270	Summer

GG_54188_c2_g1_i1	AP-1 complex subunit sigma-2	AT4G35410	Summer
GG_9948_c53_g1_i1	protein EXECUTER 2, chloroplastic	AT4G33630	Summer
GG_41580_c36_g1_i1	putative 4-hydroxy-4-methyl-2-oxoglutarate aldolase 3	AT5G56260	Summer
GG_17675_c17_g1_i2	1-aminocyclopropane-1-carboxylate oxidase homolog 1-like		Summer
GG_18067_c7_g1_i1	polymerase zeta catalytic subunit	AT1G67500	Summer
GG_41316_c71_g2_i3	polyubiquitin 3	AT5G03240	Summer
GG_40800_c78_g1_i1	30S ribosomal protein S31, chloroplastic	AT2G38140	Summer

Supplementary Table 1. *Quercus pubescens* genes or contigs differentially expressed during rain exclusion treatment.
